# Regulatory relationship between quality variation and environment of *Cistanche deserticola* in three ecotypes based on soil microbiome analysis

**DOI:** 10.1038/s41598-020-63607-2

**Published:** 2020-04-20

**Authors:** Xiao Sun, Li Zhang, Jin Pei, Lin-Fang Huang

**Affiliations:** 1 Key Research Laboratory of Traditional Chinese Medicine Resources Protection, Administration of Traditional Chinese Medicine, National administration of Traditional Chinese Medicine, Institute of Medicinal Plant Development, ChineseAcademy of Medical Sciences, Peking Union Medical College, Beijing, 100193 China; 2 Chengdu University of Traditional Chinese Medicine, Chengdu, Sichuan 611137 China; 3 College of Science, Sichuan Agriculture University, Ya’an, Sichuan 625014 China

**Keywords:** Plant ecology, Microbial ecology

## Abstract

The environment affects the composition and function of soil microbiome, which indirectly influences the quality of plants. In this study, 16S amplicon sequencing was used to reveal the differences in soil microbial community composition of *Cistanche deserticola* in three ecotypes (saline-alkali land, grassland and sandy land). Through the correlation analysis of microbial community abundance, phenylethanoid glycoside contents and ecological factors, the regulatory relationship between microbial community and the quality variation of *C. deserticola* was expounded. The metabolic function profile of soil microbiome was predicted using Tax4Fun. Data showed that the soil microbial communities of the three ecotypes were significantly different (AMOVA, P < 0.001), and the alpha diversity of grassland soil microbial community was the highest. Core microbiome analysis demonstrated that the soil microbial communities of *C. deserticola* were mostly have drought, salt tolerance, alkali resistance and stress resistance, such as Micrococcales and Bacillales. The biomarkers, namely, Oceanospirillales (saline-alkali land), Sphingomonadales (grassland) and Propionibacteriales (sandy land), which can distinguish three ecotype microbial communities, were excavated through LEfSe and random forest. Correlation analysis results demonstrated that 2′-acetylacteoside is positively correlated with Oceanospirillale*s* in saline-alkali land soil. The metabolic function profiles displayed highly enriched metabolism (carbohydrate and amino acid metabolisms) and environmental information processing (membrane transport and signal transduction) pathways. Overall, the composition and function of soil microbiomes were found to be important factors to the quality variation of *C. deserticola* in different ecotypes. This work provided new insight into the regulatory relationship amongst the environment, soil microbial community and plant quality variation.

## Introduction

*Cistanche deserticola* is a non-photosynthetic parasitic plant that can grow in dry habitats, such as sandy land and dry river ditch, as well as under stressful conditions, such as saline-alkali land^1^. This plant parasitised on the roots of *psammophyte Haloxylon ammodendron*, which shows strong resistance to harsh environmental conditions^2^. Plants rely on the beneficial interaction between roots and microorganisms to obtain nutrients, promote growth and resist external stress^3^. Meanwhile, the soil microbiome governs the biogeochemical cycling of macronutrients, micronutrients and other elements that are vital for plant growth^4^. However, the relationship between *C. deserticola* and rhizosphere soil microorganisms remains unclear. Consequently, presenting the rhizosphere soil microbiome of *C. deserticola*is necessary.

*C. deserticola* is often used in East Asia, Central Asia, North Africa and other countries as a kind of food and health care medicine to improve memory, enhance sexual function and protect the liver^5,6^. The main active ingredients of *C. deserticola* are phenylethanoid glycosides (PhGs), iridoids, lignans and polysaccharides. Amongst these components, the PhGs are the active components^7^. Previous study reported that the content of PhGs in different ecotypes of *C. deserticola* considerably differed, amongst which 2′-acetylacteoside could be used as a chemical marker to distinguish *C. deserticola* produced in Xinjiang and Inner Mongolia^1^. Studies on microbial communities have shown that microbial communities can regulate the metabolism of their hosts^8,9^. Therefore, a comparison of the relationship between microbial community and metabolites in the rhizosphere soil of *C. deserticola* in different ecotypes is urgently needed.

16S amplicon sequencing of plant rhizosphere soil samples has been performed to explore the diversity of microbial communities, providing new insights into the relationship between plants and soil microbial communities^10,11^. For example, The association of the distribution and dynamics of endophytic fungi with *C. songaricum* and *N. tangutorum*was investigated in microbiome studies using high-throughput sequencing^11^. In this study, we performed 16S rRNA amplicon sequencing to obtain the soil microbiomes of *C. deserticola* in different ecotypes. The differences of soil microbial community in various ecotypes were also compared, and the biomarkers that could distinguish the three ecotypes were excavated. Correlation analysis was then calculatedvia the key microbial community abundance, the content of PhGs and ecological factors, and the regulatory relationship was explored. Finally, the function of soil microbiomes of *C. deserticola* in different ecotypes was predicted.

## Materials and Methods

### Study site description and sampling

**Figure 1:**
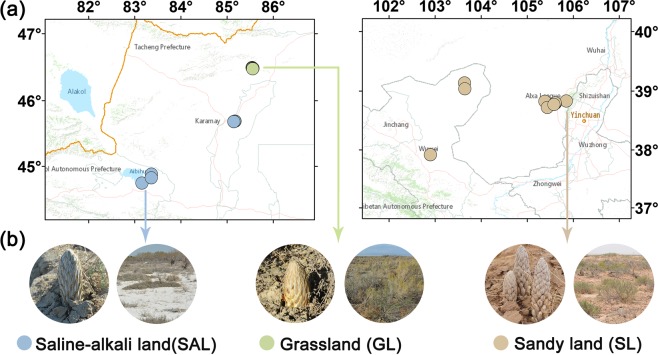
Soil sampling points map and close-up photos of plants *C. deserticola* in saline-alkali land (SAL), grassland (GL) and sandy land (SL).

According to field investigations, *C. deserticola* has three main ecotypes, including saline-alkali land, grassland and sandy land. In April, 2017, we collected soil samples representing the major ecotypes of *C. deserticola* in China. Soil samples of saline-alkali land were collected from Ebinur Lake (AB1, AB2, AB3) andBaicheng Beach (BJ1, BJ2, BJ3, BJ4, BJ5) in Xinjiang province. Grassland soil samples were taken fromTula Village (TL1, TL2, TL3, TL4, TL5) in Xinjiang province. Soil samples of Sandy land were collected from Alxa (AL1, AL2, AL3, AL4, AL5, AL6) in Inner Mongolia province and Minqin county (GS1, GS2), Changcheng county (GS3, GS4) in Gansu province. The longitude, latitude and altitude information of all sampling points are shown in Table 1 and Fig. 1. At the field site, we used a stainless steel cylindrical drill with a diameter of 5 cm to collect the soil adjacent to the succulent stem of *C. desertica* and its host, and immediately stored it in a portable refrigerator at –20 °C. After transport to the laboratory, the soil samples were passed through a 2-mm sieve to remove plant tissues, roots, rocks, etc. and stored at –20 °C in a refrigerator before further experiments.

**Table 1:** Summary of soil sample information, sequencing and statistical data of bacterial microbiome of *C. deserticola* in the three habitats.

Habitat	Sample	Origin	Longitude	Latitude	Altitude/m	Raw-tags	Clean-tags	Final-tags	OTUs
Saline-alkali land (SAL)	AB1	Ebinur Lake, Xinjiang	83.35867500	44.88165900	211.00	34987	26434	16616	1082
	AB2	Ebinur Lake, Xinjiang	83.15277000	44.74575788	199.00	42291	35078	24889	1145
	AB3	Ebinur Lake, Xinjiang	83.35642500	44.82563500	215.43	47478	36797	25491	1102
	BJ1	Baicheng Beach, Tacheng District, Xinjiang	85.17118692	45.69194949	897.00	35615	27934	18898	1236
	BJ2	Baicheng Beach, Tacheng District, Xinjiang	85.17118757	45.69195357	875.00	36004	29015	20666	1118
	BJ3	Baicheng Beach, Tacheng District, Xinjiang	85.16826932	45.69087437	875.00	45082	37422	26484	1323
	BJ4	Baicheng Beach, Tacheng District, Xinjiang	85.14605999	45.68357778	887.55	31098	25820	17213	858
	BJ5	Baicheng Beach, Tacheng District, Xinjiang	85.14610899	45.68361603	886.47	24823	20511	16371	723
Grassland (GL)	TL1	Tula Village, Xinjiang	85.54047700	46.49802700	824.76	36974	30528	18568	1229
	TL2	Tula Village, Xinjiang	85.54816200	46.49354100	797.30	45381	39005	24321	1574
	TL3	Tula Village, Xinjiang	85.55622500	46.48325600	767.32	29685	23583	14894	1332
	TL4	Tula Village, Xinjiang	85.56074299	46.48547572	788.00	26662	22196	15097	1249
	TL5	Tula Village, Xinjiang	85.55331320	46.47895172	775.98	25693	23195	15778	966
Sandy land (SL)	AL1	Alxa, Inner Mongolia	105.84898800	38.83467200	2221.87	46863	43688	32093	1491
	AL2	Alxa, Inner Mongolia	105.38391600	38.82816300	1316.97	34046	32927	24225	1357
	AL3	Alxa, Inner Mongolia	105.43757700	38.72539100	1307.60	33594	31880	25639	1174
	AL4	Alxa, Inner Mongolia	105.61283393	38.79440892	1321.46	37147	33047	24047	1169
	AL5	Alxa, Inner Mongolia	105.60756354	38.80077933	1321.52	34622	31691	23262	1226
	AL6	Alxa, Inner Mongolia	105.58088264	38.77560342	1321.89	42533	38015	23537	1257
	GS1	Minqin county, Gansu	103.63953901	39.13820780	1347.00	48337	47235	39126	1176
	GS2	Minqin county, Gansu	103.64362489	39.04050036	1356.21	48112	46445	37221	1312
	GS3	Changcheng county, Gansu	102.89698463	37.91689645	1417.79	23731	23558	19546	851
	GS4	Changcheng county, Gansu	102.89698463	37.91689645	1419.00	23565	23389	18947	823

### DNA Extraction and *16S rRNA* Sequencing

Soil DNA was extracted using the PowerSoil DNA Isolation Kit (MoBio Laboratories, Carlsbad, CA) following the manual^12^. The purity and quality of the genomic DNA were checked by 0.8% agarose gels electrophoresis and nanodrop.

The V3-V4 hypervariable region of bacterial 16S rRNA gene was amplified with the primers 338F (ACTCCTACGGGAGGCAGCAG) and 806R (GGACTACHVGGGTWTCTAAT)^13^. For each soil sample, a 10-digit barcode sequence was added to the 5′ end of the forward and reverse primers (Allwegene Company, Beijing)^12^. The PCR was performed on a Mastercycler Gradient (Eppendorf, Germany) using 25 μl reaction volumes containing 12.5 μl KAPA 2G Robust Hot Start Ready Mix, 1 µl forward primer (5 µM), 1 µl reverse primer (5 µM), 5 µl DNA (total template quantity is 30 ng) and 5.5 µl H_2_O. The cycling parameters are as follows: 95 °C for 5 min, followed by 28 cycles of 95 °C for 45 s, 55 °C for 50 s and 72 °C for 45 s, with a final extension at 72 °C for 10 min. Three PCR products per sample were pooled to mitigate reaction-level PCR biases. The PCR products were purified using a QIAquick Gel Extraction Kit (QIAGEN, Germany) and then quantified using real-time PCR^14^.

Deep sequencing was performed on a MISeq platform at the Allwegene Company (Beijing). After the run, image analysis, base calling and error estimation were performed using Illumina Analysis Pipeline Version 2.6^14^.

### Data analysis

The raw data were firstly screened, and sequences were removed based on the following considerations: sequences shorter than 200 bp with low quality score (≤20) and contained ambiguous bases or did not match the primer sequences and barcode tags^15^. Qualified reads were separated using the sample-specific barcode sequences and trimmed with Illumina Analysis Pipeline Version 2.6. Then, the datasets were analysed using QIIME. The sequences were clustered into operational taxonomic units (OTUs) at a similarity level of 97%^16^ to generate rarefaction curves and calculate the richness and diversity indices. The Ribosomal Database Project Classifier tool was used to classify all sequences into different taxonomic groups^15^. Clustering analyses were performed based on the OTU information from each sample using R to examine the similarity between different samples^17^. The UniFracdistances matrix between microbial communities from each sample were calculated using the Tayc coefficient and represented as an unweighted pair-group method with arithmetic mean clustering tree, which describes the dissimilarity (1-similarity) amongst the multiple samples^18^. A Newick-formatted tree file was also generated through this analysis. Alpha diversity was applied in the analysis of the complexity of species diversity for a sample using four indices, including Chao1, observed species, phylogenetic diversity (PD) whole tree and Shannon diversity index. These indices were calculated using the QIIME software (Boulder, CO, USA) in Python (v.1.8.0) (La Jalla, CA, USA)^19^. Beta diversity analysis was used to evaluate differences of samples in terms of species complexity. Beta diversity was calculated using the principal coordinate analysis (PCoA) and cluster analysis in QIIME^20^. The analysis of molecular variance (AMOVA) was performed using mothur. EdgeR was used to calculate the OTU difference between groups. Heatmap.2 was used to draw the heat map, whilst Ggplot was used to draw the Manhattan map.

### Determination of biomarker and core microbiome

The linear discriminant analysis (LDA) and random forest (RF) methods in the Microbiome Analyst website^21^ was used to determine the biomarker microbiome. The website firstly performs non-parametric factorial Kruskal–Wallis sum–rank test to identify features with significant differential abundance considering the experimental factor or class of interest, followed by LDA to calculate the effect size of each differentially abundant features^21^. The features are considered significant based on their adjusted p-value. The default adj.p-value cutoff = 0.05. RF analysis is performed using the randomForest package5. RF is a supervised learning algorithm that is suitable for high-dimensional data analysis. This method utilises an ensemble of classification trees, each of which is grown via random feature selection from a bootstrap sample at each branch^22^. Core microbiome analysis was adopted from the core function in the R package microbiome by Microbiome Analyst (https://www.microbiomeanalyst.ca/MicrobiomeAnalyst/home.xhtml).

### Correlation analysis of key microbial communities, *PhGs* content and ecological factors

The contents of seven phenylethanoid glycosides (PhGs) of the three ecotypes of *C. deserticola*, namely, echinacoside, cistanoside A, acteoside, isoacteoside, 2′-acetylacteosid, tubuloside A and cistanoside F, were determined through HPLC. The chromatographic conditions involve a Waters C18 column (150 mm × 3.9 mm, 4.6 μm), and the mobile phase comprises acetonitrile and 0.2% formic acid. The chromatographic settings were as follows: 0–10 min, 10%→15% A; 10–30 min, 15%→40% A. The flow rate was 1 mL/min. The absorption wavelength, injection volume and column temperature were 330 nm, 10 μL and 27 °C, respectively. The methodology study refers to the preliminary experimental work of the reference group^1^.

This study collected 16 meteorological stations near the three ecological types of *C. deserticola* (http://data.cma.cn/): Xinjiang Bole 51238, Tacheng 51133, Tori 51241, Karamay 51243, Buxail 51156, Yumin 51137, Emin 51145, Gansu Minqin 52681, Yongchang 52674, Wuwei 52679, Gulang 52784, Inner Mongolia Suikou 53419, Hangjinhouqi 53420 and Wuhai 53512. Data of seven climatic factors from 1981–2010 served as the climatological factor data for subsequent correlation analysis.

Redundancy analysis of differential metabolites and bioclimatic factors was performed by using Canoco 5 software^23^. Pearson correlation coefficient was calculated for biomarker microbiome abundance, compound content and ecological factor data integration. In this study, the log_2_ data conversion was uniformly performed before the analysis. SPSS was used to calculate the Pearson correlation coefficient of the six biomarkers, six core microbiomes, seven main active components of *C. deserticola* and the ecological factors in the three habitats, and the screening standard was as follows: pearson correlation coefficient (r) >0.5 and *p* value < 0.05. The relationships amongst the above factors were visualised using Cytoscape^24^ (The Cytoscape Consortium, San Diego, CA, USA, version 3.7.0) and pheatmap (R package).

### Prediction of the microbial functional profiles of the microbiome

Tax4Fun^25^ (R package, http://tax4fun.gobics.de/) was used to predict the microbial functional profiles of microbiomes in the soil samples. The OTU Biom table of soil microbiome was used as an input file for the metagenome imputation of *C. deserticola* soil samples. Then, the predicted gene class abundances were analysed at the KEGG Orthology (KO) group level 3. The results from Tax4Fun were analysed in doBy (R package)^26^.

## Results

### Microbial community composition

The 16S rRNA sequencing resulted in 834,323 raw reads, amongst which 522,929 passed the quality and length filtering. The data set comprised 20,511–48,337 (mean: 33,994) sequences per sample. The high-quality reads were clustered using >97% sequence identity into 3541 microbial OTUs (Table 1).

**Figure 2:**
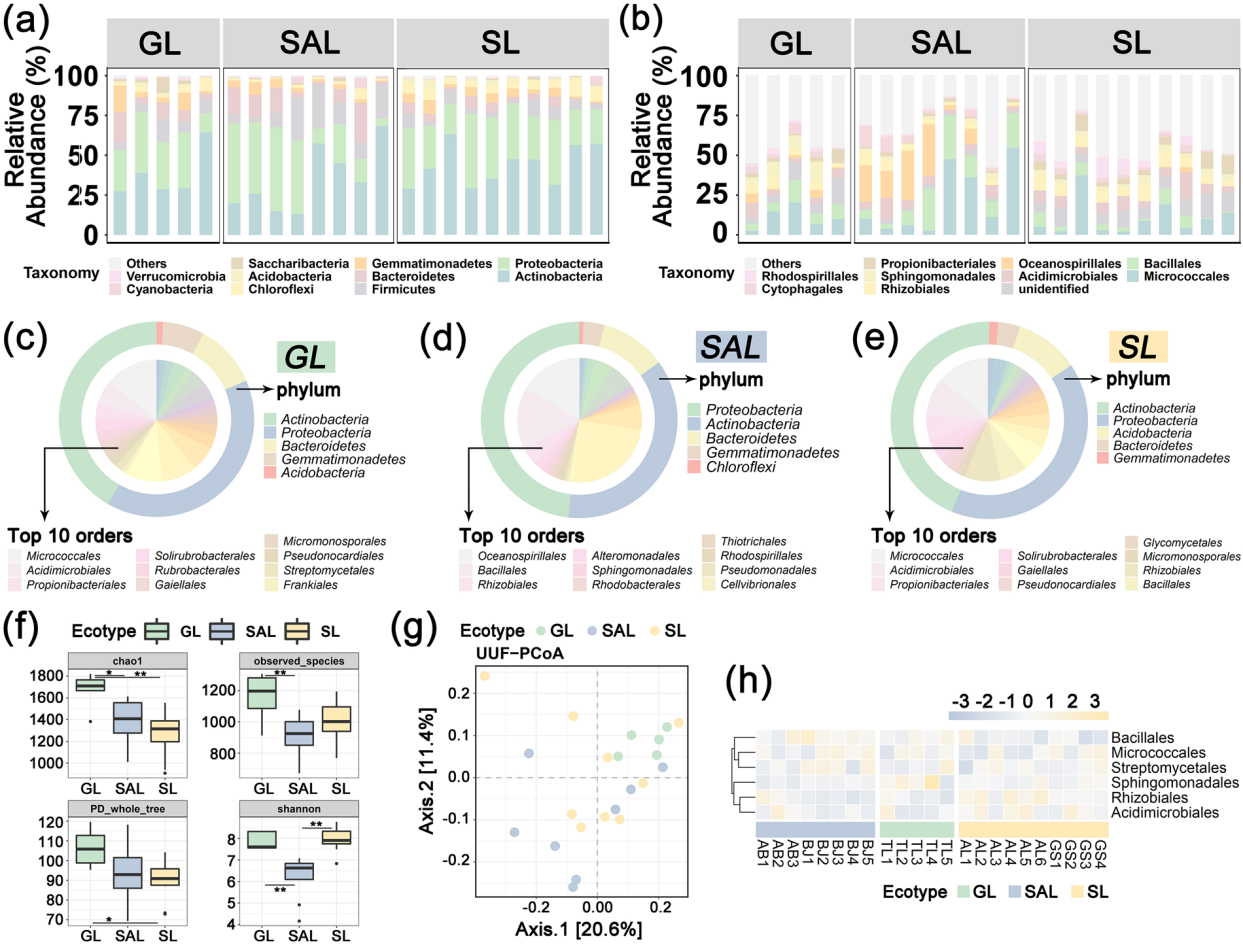
Classification of the microbial community composition across the three ecotypes of *C. deserticola*. (**a**) Histograms of phyla abundances. (**b**) Histograms of orders abundances. (**c**) Pie chart of the top 10 microbial orders and their phylum-level species in grassland. (**d**) Pie chart of the top 10 microbial genera and their phylum-level species in saline-alkali land. (**e**) Pie chart of the top 10 microbial genera and their phylum-level species in sandy land. (**f**) Within-sample diversity (α-diversity). *represents P value < 0.05; **represents P value < 0.01. (**g**) PCoA plot based on the unweighted UniFrac distance matrix of the 16S rRNA gene amplicons. (**h**) Heatmap of core microbial abundance of three ecotypes of *C. deserticola*. SAL: Saline-alkali land, GL: Grassland and SL: Sandy land.

The microbial community was generally classified into 19 phyla, 46 classes and 87 orders (Figs. 2a,b and S1–S3). At the phylum level, Actinobacteria (35%), Proteobacteria (31%) and Firmicutes (15%) were dominant in saline-alkali land. The grassland was dominated by Actinobacteria (37%), Proteobacteria (29%) and Bacteroidetes (9%). In sandy land, Actinobacteria (42%), Proteobacteria (33%) and Firmicutes (6%) were dominant. At class level, Actinobacteria (25%), Gammaproteobacteria (21%) and Bacilli (15%) were dominant in saline-alkali land, whilst Actinobacteria (22%), Alphaproteobacteria (16%) and Bacilli (8%) were dominant in grassland. In sandy land, Actinobacteria(23%), Alphaproteobacteria (20%) and Gammaproteobacteria (9%) dominated. At the order level (Fig. 2c–e), Micrococcales (22%), Oceanospirillales (15%) and Bacillales (15%) were dominant in saline-alkali land. Micrococcales (11%) and Bacillales (8%) dominated in grassland. Thus, the most dominant bacterial orders were Micrococcales (10%) and Acidimicrobiales (7%).

### Diversity of soil bacteria in the three ecotypes of C. deserticola

The measures of within-sample diversity (α-diversity) revealed a diversity gradient amongst the three habitats (Fig. 2f and Table S1). The alpha diversity of the soil bacterial microbial community of each sample was estimated using the community richness (Chao 1, which was expressed as the projected total number of OTU in each sample), observed species, PD whole tree and Shannon diversity index. The observed species, Chao 1 and PD whole tree index suggested that grassland soil communities had the highest α-diversity, whilst the α-diversity of saline-alkali and sandy land soils is similar. The results of therarefaction curves (Fig. S1) are similar to the above results.

The AMOVA results (Table S2) showed that p < 0.001, which indicates a significant difference amongst the three habitats. The bray distance diversity tree clustering results (Fig. S2) of the three habitat soil samples demonstrated that the saline-alkali soil samples were closely clustered, and the distance between the grassland and sandy soil samples was close. The results of the unconstrained PCoAs of unweighted UniFrac distance 2D plots (Fig. 2g) showed that the different soil samples of bacterial microbial community from different habitats were well-clustered.

### Comparison of soil bacterial communities of the three ecotypes of C. deserticola

**Figure 3:**
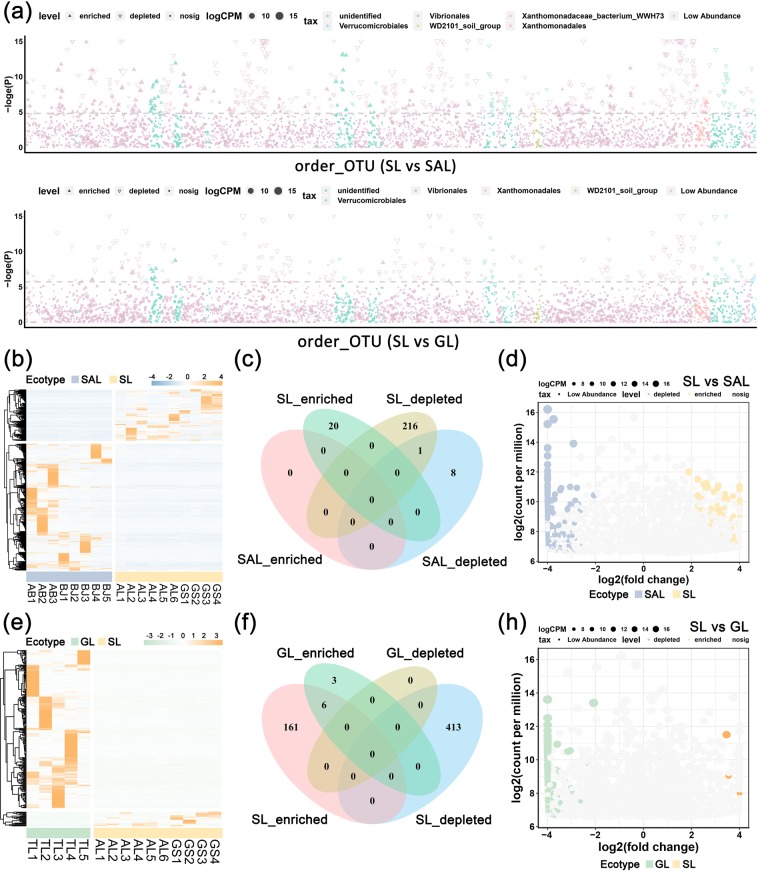
Differential microbial profiles of three ecotypes of *C. deserticola*. (**a**) Manhattan plots showing enriched and depleted OTUs in saline-alkali land vs. sandy land or grassland vs. sandy land at order level. The dashed line corresponds to the false discovery rate-corrected P value threshold of significance (α = 0.05). The size of the point represents the relative abundance of the OTUs. The point identifies the type of changes, the shape of the solid triangle represents increased enrichment, hollow triangles represent the cut depleted and solid dots indicate no significant difference. (**b**) Heatmap of the OTU difference between saline-alkali land and sandy land; (**c**) Venn diagram of the OTU difference between saline-alkali land and sandy lands. (**d**) Volcano map showing differential microbial orders between saline-alkali land and sandy land. (**e**) Heat map of the OTU difference between grassland and sandy land; (**f**) Venn diagram of the OTU difference between grassland and sandy lands. (**g**) Volcano map showing differential microbial orders between grassland and sandy land. SAL: Saline-alkali land, GL: Grassland and SL: Sandy land.

Different OTU abundance heatmapsand volcano map(Fig. 3b,e,d,h and Table S3) indicated that compared with sandy land samples, 24 and 17 OTUs were respectively enriched and depleted in saline-alkali land at order level. In addition, compared with sandy land samples, 8 and 11 OTUs were respectively enriched and depleted in the grassland. The Venn diagram (Fig. 3c,f) illustrated that 216 OTUs were depleted in sandy land, 8 were depleted in saline-alkali land and 1 OTU overlapped between saline-alkali and sandy lands. Furthermore, 3 OTUs were enriched in grassland, whilst 161 were enriched in sandy land, including 6 OTUs that overlapped between grassland and sandy land. The Manhattan plots (Fig. 3a) demonstrated that Xanthomonadales were more enriched and depleted in saline-alkali land than those in sandy land, whilst Xanthomonadaceae bacterium WWH73, WD2101 soil group, Vibrionales and Verrucomicrobiales were significantly depleted at order level. Comparing grassland with sandy land, Xanthomonadales, WD2101 soil group, Vibrionales and Verrucomicrobiales were significantly depleted.

### Determination of biomarker and core microbiome in the three ecotypes of *C. deserticola*

**Figure 4:**
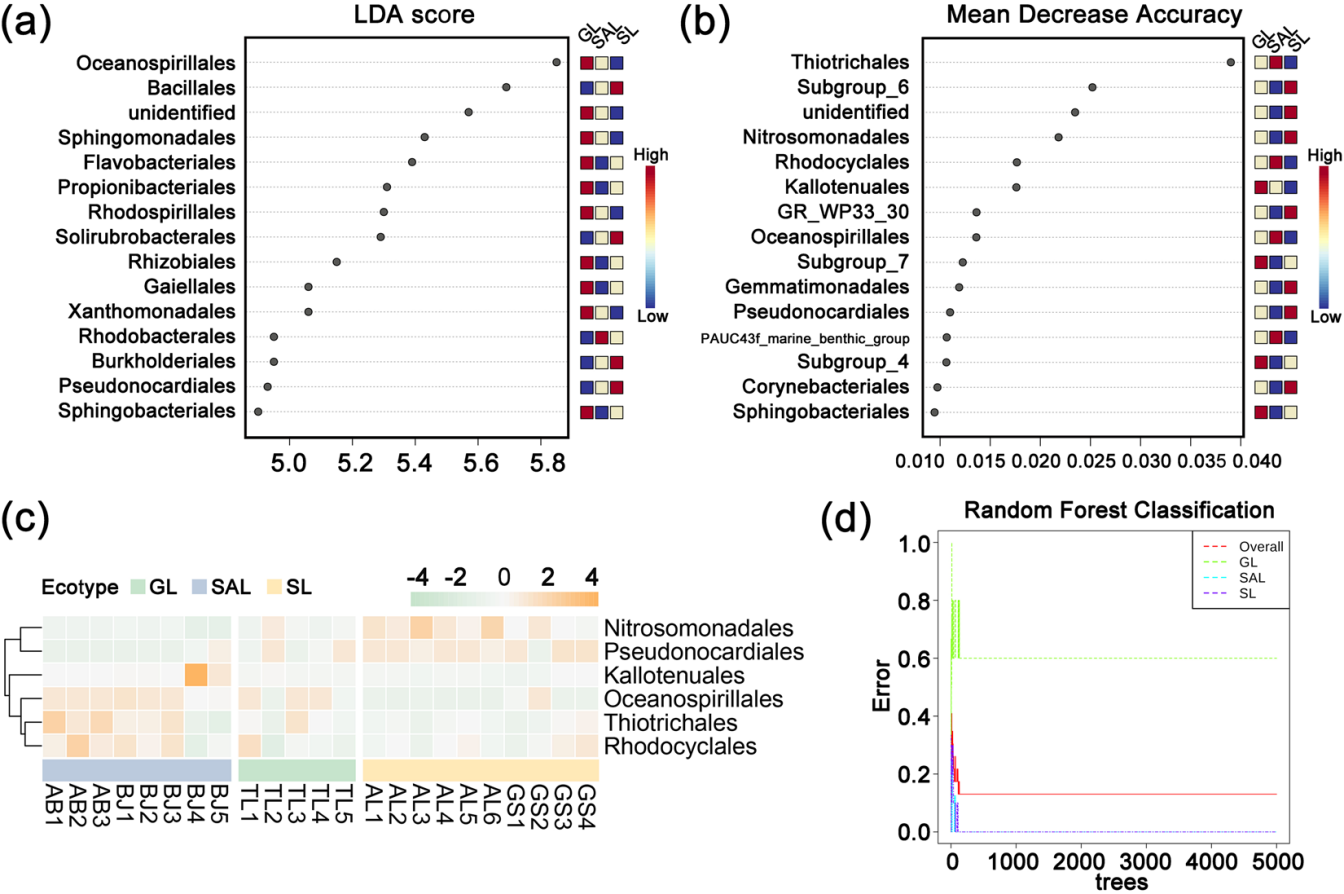
(**a**) Graphical summary at order level in group sample type of the top 15 biomarkers of *C. deserticola* soil in the three habitats. (**b**) Significant features identified by Random Forest. The features are ranked by the mean decrease in classification accuracy when they are permuted. (**c**) Heatmap of biomarkers abundance of three ecotypes of *C. deserticola*. (**d**) Cumulative error rates obtained through RF classification. The overall error rate is represented by the red line, whilst the error rates for each class are represented by the green, blue and purple lines. SAL: Saline-alkali land, GL: Grassland and SL: Sandy land.

The LEfSe and RF (Fig. 4a,b,d) methods were used to identify features with significant differential abundance across the soil samples, calculate the effect size of each differentially abundant features and determine the biomarker bacterial microbiome in the three habitats at order level. The results presented in Table S4 revealed that the LDA score of Oceanospirillales, Bacillales and Flavobacteriales was the highest amongst those in the saline-alkali land, whilst that of Sphingomonadales, Gaiellales, Rubrobacterales, Burkholderiales and Sphingobacteriales was the highest amongst those in grassland. The LDA score of Propionibacteriales, Rhodospirillales, Solirubrobacterales, Rhizobiales, Xanthomonadales and Pseudonocardiales were dominant in sandy land. With the exclusion of undefined and duplicated orders, these OTUs were classified into six orders, and their abundances were drawn in a heatmap (Fig. 4c).

The persistence method was adopted from the core function in the R package microbiome to identify the core microbiome in the three ecotypes of *C. deserticola*. This core bacterial microbiome contained six orders, including Micrococcales, Bacillales, Rhizobiales, Acidimicrobiales, Streptomycetales andSphingomonadales. With the exclusion of undefined and duplicated orders, these OTUs were classified into six orders, and their abundances were drawn in a heatmap (Fig. 2h).

### Correlations amongst key microbial community abundance, *PhGs* content and ecological factors

**Figure 5:**
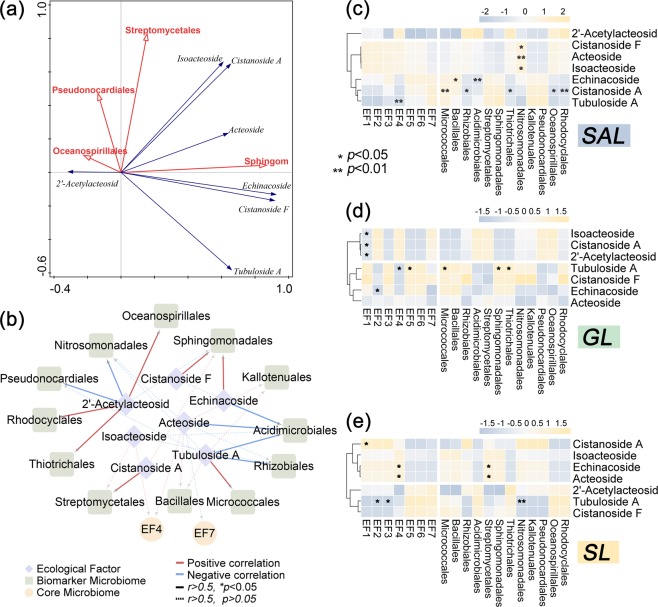
Correlation analysis based on key microbiome (six biomarkers and six core microbiome), seven active components and ecological factors. (**a**) RDA plot of overall key microbes, active components and ecological factors by Canoco 5. (**b**) Network for correlation analysis of overall key microbes, active components and ecological factors. (**c**) Heatmap for correlation analysis of key microbes, active components and ecological factors in saline-alkali land. (**d**) Heatmap for correlation analysis of key microbes and ecological factors in grassland. (**e**) Heatmap for correlation analysis of key microbes and ecological factors in sandy land.*represents *p* value < 0.05; **represents *p* value < 0.01. SAL: Saline-alkali land, GL: Grassland and SL: Sandy land.

The redundant analysis of the core, biomarker microbiome abundance, PhGs content and ecological factors was performed at the order level, and reanalysis was performed on the basis of effects. The adjusted interpretation of variance was 82.70% (Table S7). The Sphingomonadales explained 45.7% of *PhGs content* (p = 0.002). The Pseudonocardiales explained 22.4% of *PhGs content* (p = 0.01). The 2′-acetylacteosid was significantly positively correlated with Pseudonocardiales and Oceanospirillalesand negatively correlated with Sphingomonadales (Fig. 5a). The echinacoside was significantly positively with Sphingomonadales.

Correlation analysis was conducted for biomarker abundance, PhG contents (Fig. S3 and Table S5) and ecological factors (Table S6). The results of the correlation networks (Fig. 5b) revealed that 2′-acetylacteosid was significantly positively correlated with Oceanospirillales, Rhizobiales and Thiotrichales, whilst negatively correlated with Pseudonocardiales and Nitrosomonadales in all soil samples. The heatmap (Fig. 5c–e) revealed that tbuloside Awas negatively correlated with Average annual water vapor pressure in saline-alkali land. Meanwhile, cistanoside Awas significantly positively correlated with Micrococcales and negatively correlated with Rhizobiales in saline-alkali land (Fig. 5c). In grassland,2′-acetylacteosidwas negatively correlated with average annual temperature (Fig. 5d). In sandy land, tubuloside A wasnegatively correlated with Nitrosomonadales (Fig. 5e).

### Predictive function of bacterial microbiome in the three ecotypes of *C. deserticola*

**Figure 6:**
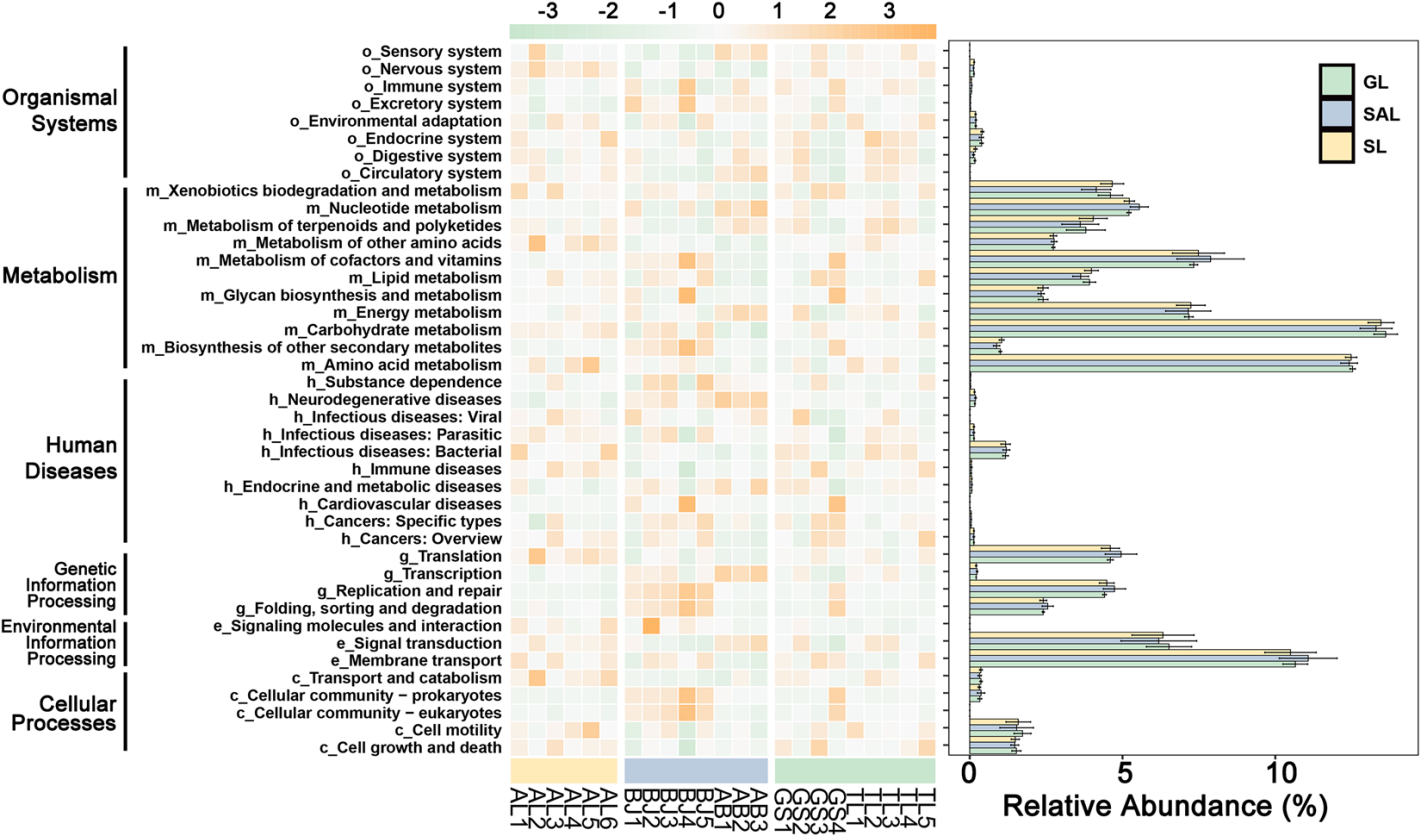
The heatmap of normalized relative abundance of imputed functional profiles of KOs assigned to KEGG pathways within *C. deserticola*soil in three habitats bacterial microbiome using PICRUSt grouped into level-3 functional categories.

The functional profiles of bacterial microbiome were predicted based on the 16S rRNA gene copy number of deciphered bacterial taxa using Tax4Fun according to the KEGG Ortholog groups (KOs). The results of functional prediction (Fig. 6) demonstrated that the functional metabolisms of soil microbiomes in the three ecotypes of *C. deserticola* were identical. Amongst the metabolisms, carbohydrate, amino acid, co-factors and vitamin and energy metabolisms were abundant. Membrane transport and signal transduction were also abundant in environmental information processing.

## Discussion

Our previous work demonstrated that psbA-trnH sequence and 2′-acetylacteosid can be used as molecular and chemical markers to distinguish *C. desertica* from Xinjiang and Inner Mongolia^27^. With field investigations, we found that *C. desertica* inhabits mainly in three types of habitats, including saline-alkali land by EbinurLake, sandy land around Alxa League and intermediate desert grasslands. The metabolic profiles of three ecotype *C. desertica* also showed that 2′-acetylacteosid can be used as a chemical marker to distinguish the three ecotypes^1^. We discussed the variation of *C. desertica* quality and its formation mechanism from the dimensions of heredity, metabolism and climatic factors. Therefore, from a micro perspective, the correlation network analysis of microbiome abundance, PhGs contents and ecological factors was conducted to elucidate the feature of soil microbial community of the three ecotypes of *C. deserticola* and their relationship with the quality variation.

### Characteristics of soil microbial communities in three ecotypes of *C*. deserticola

The composition and structure of rhizospheric microbial communities vary across different soil types^28^. Table 1 indicates that habitat is the main factor that influences the difference in soil microbial community composition. The α-diversity (Fig. 2e) suggested that the richness of grassland soil microbiomes was the highest due to its higher vegetation richness and precipitation than that in saline-alkaline and sandy lands. Moreover, the average annual temperature was lower in the former than that in the latter two (Table S6). Salt, alkali and drought stresses exist in saline-alkali soil, and drought stress is present in sandy land. High temperature and low precipitation were also observed. These factors contribute to the lower microbial abundance of saline-alkali and sandy land soils than that of grassland soil.

Micrococcales and Bacillales are the coremicrobiomes of the three ecotypes (Fig. 2h). Micrococcales are common in soil and water and are generally salt-tolerant that can be grown in 5% NaCl^29^. Many studies have found that Micrococcales are enriched in arid environments^30^ and in halophytes^31^. Bacillales are an order of gram-positive bacteria placed within the Firmicutes^32^. Representative genera include Bacillus, Listeria and Staphylococcus. Bacillales have strong resistance to heat, drying, radiation, chemical disinfectants and other physical and chemical factors, which may be related to the unique high content of pyridine dicarboxylic acid. Bacillales is resistant to high temperature, strong acid, strong alkali and high and low oxygen content, which may be attributed to its high pyridine dicarboxylic acid content. Bacillales moisture can provide substantially high strength of natural material polyglutamic acid and soil protective film and prevent the loss of fertilizer and water. Moreover, Bacillalescan produce rich metabolic products to synthesise a variety of organic acids, enzymes, physiological activities and other substances, as well as a variety of other nutrients that can be easily utilised^33^. The common environmental characteristics of three ecotypes of *C. desertica* are drought and soil desertification. The soil around Ebinur Lake is also accompanied by saline-alkali stress. This may be the reason that the core soil microbial communities of three ecotypes have certain characteristics of drought, salt tolerance, alkali resistance and stress resistance.

### Microbial factors affecting the variation of PhGs in three ecotypes of *C. deserticola*

Fig. S3 shows a box diagram of the PhG content of the three ecotypes of *C. deserticola*. The figure reveals that the 2′-acetylacteosid content is higher in saline-alkali land than in grassland and sandy land, which is consistent with previous results^1^. Redundant analysis and association network results shows that Oceanospirillales is significantly positively correlated with 2′-acetylacteosid. Oceanospirillales, the highest biomarker in terms of LDA score in saline-alkali land, are specifically enriched. These bacteria are metabolically and morphologically diverse, some of which can grow in the presence of oxygen whilst others require an anaerobic environment^34^. Oceanospirillales are an order of Proteobacteria comprising two families. Marine spirillum is often an endosymbiont of bone-eating worms (Osedax)^35^. Most Oceanospirillales prefer or require high salt concentrations to grow. Despite their growth in diverse niches, Oceanospirillales derive energy from the breakdown of various organic products. Therefore, the high salinity and alkalinity are the main reasons for the enrichment of Oceanospirillales in saline-alkali land soil. This result strongly suggests that the highest content of 2′-acetylacteosid in saline-alkali is related to the enrichment of Oceanospirillales. However, the regulatory relationship between Oceanospirillales and 2′-acetylacteosid is still blank, and further research is needed.

The overall contents of the seven PhGs are the highest in the grassland, amongst which echinacoside is the dominant PhGs. Echinacoside is significantly positively correlated with Sphingomonadales, which is a sequence within the alpha-proteus and constitutes the family of Erythrobacteraceae and Sphingomonadaceae. Both families are common in nature, especially in soils, oceans and freshwater^36^. Sphingomonadales has a wide range of metabolic capacity for aromatic compounds, and some strains can synthesise valuable extracellular biopolymers^37^. All previously known members of the class Sphingomonas are aerobic and chemically organic. The only exception is the facultative anaerobic ethanol fermenter, which is used to produce fermented beverage pulp. Certain species of the genus Rhodobacter, porphyrin and Staphylococcus aureus, as well as certain species of the genus Sphingomonas, have chlorophyll a and are therefore optional photo-organotroph (energy generated via photosynthesis)^38^. The best quality of *C. deserticola* in grassland may be due to the rich microbial community diversity and metabolic-related functions of the biomarker (Sphingomonadales). This finding provides new insight into the study on the quality variation of *C. deserticola* in different ecotypes.

### Prediction metabolic function profiles of soil microbiomes of the three ecotypes *C. deserticola*

The metabolic function profiles (Fig. 6 and File S1) of soil microbiomes of *C. deserticola* in the three ecotypes were demonstrated for the first time in this study. In terms of metabolism, carbohydrate metabolism (starch and sucrose metabolism,ko00500; amino sugar and nucleotide sugar metabolism, ko00520) and amino acid metabolism (arginine and proline metabolism, ko00330; glycine, serine and threonine metabolism, ko00260) are highly enriched in microbiomes. Carbohydrate metabolism is responsible for the formation, breakdown, and conversion of carbohydrates in the body. Carbohydrates are the basis of many important metabolic pathways^39^. Carbohydrates such as glucose are part of multiple metabolic pathways across species. Carbohydrates are synthesized by plants from the atmosphere through photosynthesis and can be used as substrates for cellular respiration^40^. Plants and microorganisms absorb ammonia, ammonium salt, nitrite, nitrate and other inorganic nitrogen from the environment to synthesise proteins and nitrogen-containing substances. Some microbes can convert N_2_ from air into ammonia nitrogen to synthesise amino acids^41^. The metabolic function of soil microbiomes was enriched in the primary metabolism. This enrichment suggests that the microbiomes can provide nutrition to plants and promote their growth under drought and other stresses.

Metabolic function profiles also showed that in environmental information processing, membrane transport (ABC transporters, ko02010) and signal transduction (two-component system, ko02020) are highly enriched in microbiomes. Membrane transport is a collection of mechanisms that regulate the passage of solutes, such as ions and small molecules, through a biofilm, which is a bilayer of lipids embedded in proteins. The regulation of crossing membranes is attributed to the permeability of selective membranes, a characteristic of biofilms that enables the separation of substances with different chemical properties. In other words, these membranes might be permeable to some substances but not to others^42^. Amongst these membranes, ABC transporter pathway was highly enriched in soil microbiomes. ATP-binding box (ABC) transporters are universally existed in microorganisms such as bacteria and is one of the biggest protein families known today. These transporters bind ATP hydrolysis to participate in the active transport of multifarious substrates such as ions, peptides, lipids, drugs, sugars, proteins and sterols. The structure of ABC transporters in prokaryotes usually comprises three parts. Generally, two intact membrane proteins each have six transmembrane fragments: two peripheral proteins that bind and hydrolyze ATP, and one peripheral (or lipoprotein) substrate of a binding protein. As observed in the genomes of many bacteria and archaea, many genes of these three components form operons^43^. Drought, salinity and alkali stress promoted the membrane transport function, especially the improvement of active transport function of soil microbiomes.

Signal transduction is a process in which a chemical or physical signal is transmitted through a cell as a series of molecular events. The most common is protein kinase-catalyzed protein phosphorylation. The two-component system is a signaling pathway that regulates many bacterial characteristics, such as toxicity, pathogenicity, symbiosis, motility, nutrient absorption, production of secondary metabolites, metabolic regulation, and cell division. These systems regulate physiological processes based on environmental or cellular parameters, enabling them to adapt to changing conditions^44^. The signal transduction of soil microbiomes was promoted by drought or saline-alkali stress.

## Conclusion

This study is the first to present the soil microbiomes of the three ecotypes of *C. deserticola*. The following conclusions are obtained: (1) soil microbial community in grassland is the most abundant amongst the three habitats. (2) The biomarkers of the three ecotypes were also determined: Oceanospirillales (saline-alkali land), Sphingomonadales (grassland) and Propionibacteriales (sandy land). (3) Core microbiome analysis demonstrated that the soil microbial communities of *C. deserticola* were mostly have drought, salt tolerance, alkali resistance and stress resistance, such as Micrococcales and Bacillales. (4) The correlation analysis demonstrated that 2′-acetylacteoside is positively correlated with Oceanospirillales and echinacoside is significantly positively correlated with Sphingomonadales. (5) Tax4Fun predicts that the metabolic function profiles of three ecotypes of soil microbiome are enriched in metabolism and environmental information processing.

## Supplementary information

Supplementary Figure and Table.

Supplementary Dataset 1.
