# Supplementary material for: Regulatory relationship between quality variation and environment of Cistanche deserticola in three ecotypes based on soil microbiome analysis
Source: Sci Rep. 2020 Apr 20;10:6662. doi: 10.1038/s41598-020-63607-2 (PMC7170941; doi:10.1038/s41598-020-63607-2)
Supplement: Supplementary file 1 — Supplementary Figure and Table. [file 41598_2020_63607_MOESM1_ESM.docx]

**Regulatory relationship between quality variation and environment of *Cistanche deserticola* in three ecotypes based on soil microbiome analysis**

Xiao Sun^1^, Jin Pei^2,*^, Li Zhang^3,*^, Lin-Fang Huang^^[[1]](#footnote-1)^,*^

^1^ *Key Research Laboratory of Traditional Chinese Medicine Resources Protection, Administration of Traditional Chinese Medicine, National administration of Traditional Chinese Medicine, Institute of Medicinal Plant Development, Chinese* *Academy of Medical Sciences, Peking Union Medical College, Beijing 100193, China*

^2^ *Chengdu University of Traditional Chinese Medicine, Chengdu, Sichuan, 611137, China*

^3^ *College of Science, Sichuan Agriculture University, Ya’an, Sichuan, 625014, China*

Xiao Sun: [934305103@qq.com](mailto:934305103@qq.com) ORCID: 0000-0001-9169-3356

*Correspondence:

Lin-Fang Huang: [lfhuang@implad.ac.cn](mailto:lfhuang@implad.ac.cn); [15801545922@139.com](mailto:15801545922@139.com);

Jin Pei: [peixjin@163.com](mailto:peixjin@163.com)

Li Zhang: [zhangli@sicau.edu.cn](mailto:zhangli@sicau.edu.cn)

**Supplementary**

| Table S1 Alpha diversity summary of bacterial microbiome of *C. deserticola* in three ecotypes | | | | | |
| --- | --- | --- | --- | --- | --- |
| Habitat | Sample ID | Chao1 | Observed species | PD whole tree | Shannon |
| SAL | AB1 | 1605.072969 | 990.3 | 92.676873 | 6.644652952 |
|  | AB2 | 1347.327421 | 924 | 93.141654 | 7.073935647 |
|  | AB3 | 1337.307681 | 870.7 | 106.047315 | 6.839296626 |
|  | BJ1 | 1613.608505 | 1077.9 | 118.178886 | 6.487432472 |
|  | BJ2 | 1468.485308 | 926.3 | 89.041053 | 4.917014862 |
|  | BJ3 | 1538.634883 | 1033.4 | 99.97164 | 6.604328069 |
|  | BJ4 | 1094.826668 | 790.6 | 76.924028 | 6.836887914 |
|  | BJ5 | 1011.193134 | 671.7 | 69.34241 | 4.165364612 |
| GL | TL1 | 1666.92425 | 1086.3 | 98.820104 | 7.532966118 |
|  | TL2 | 1765.494552 | 1306.7 | 119.62852 | 8.329422813 |
|  | TL3 | 1819.562989 | 1282 | 112.7602 | 7.55506126 |
|  | TL4 | 1710.074057 | 1197.5 | 105.848337 | 8.314928928 |
|  | TL5 | 1382.767886 | 913 | 95.299445 | 7.601576546 |
| SL | AL1 | 1555.295577 | 1194.8 | 104.237935 | 8.402833327 |
|  | AL2 | 1471.465014 | 1175.1 | 97.925438 | 8.760790809 |
|  | AL3 | 1284.534618 | 989.2 | 91.774517 | 6.829425926 |
|  | AL4 | 1315.105148 | 978.1 | 87.057981 | 7.86711945 |
|  | AL5 | 1407.883253 | 1044.3 | 91.077385 | 8.103884275 |
|  | AL6 | 1329.526287 | 1113.6 | 90.677863 | 8.485852588 |
|  | GS1 | 1167.84913 | 927 | 89.009267 | 7.713566227 |
|  | GS2 | 1315.122637 | 1015.2 | 97.165546 | 7.935199442 |
|  | GS3 | 937.6705603 | 784.5 | 72.879613 | 7.838456142 |
|  | GS4 | 907.4770155 | 768.6 | 73.461051 | 7.489380705 |

Table S2 AMOVA results

| SAL-GL-SL | Among | Within | Total |
| --- | --- | --- | --- |
| SS | 2.13192 | 4.25853 | 6.39046 |
| df | 3 | 19 | 22 |
| MS | 0.710641 | 0.224133 |  |
| Fs: | 3.17062 |  |  |
| p-value: <0.001* |  |  |  |

Note: Analysis of molecular variance (AMOVA) showing the partitioning of genetic variation within and between varieties (df = degree of freedom, SS = sum of squares, MS mean squares, Fs = F test value, P is based on 9999 permutations)

| Table S3 OTU difference table of the different habitats at order level | | | | | | | | |
| --- | --- | --- | --- | --- | --- | --- | --- | --- |
| OTU | logFC | logCPM | LR | P Value | sig | level | neglogp | tax |
| **GL vs SAL** | | | | | | | | |
| OTU_345 | 3.850750152 | 9.664803 | 16.27338 | 5.48287E-05 | 1 | enriched | 9.811296135 | Xanthomonadales |
| **SL vs GL** | | | | | | | | |
| OTU_558 | -6.67124178 | 7.708416 | 24.28854 | 8.29311E-07 | -1 | depleted | 14.00267037 | Xanthomonadales |
| OTU_268 | -3.88372483 | 8.356257 | 12.26564 | 0.000461376 | -1 | depleted | 7.68129801 | Xanthomonadales |
| OTU_2878 | -3.85980307 | 6.66088 | 9.372241 | 0.00220296 | -1 | depleted | 6.11795317 | Xanthomonadales |
| OTU_1098 | -4.75608754 | 6.916452 | 12.02344 | 0.000525358 | -1 | depleted | 7.55143164 | WD2101_soil_group |
| OTU_1694 | -4.23164734 | 7.008551 | 12.65112 | 0.000375341 | -1 | depleted | 7.88767646 | WD2101_soil_group |
| OTU_2194 | -4.11336548 | 6.661234 | 10.42484 | 0.001243316 | -1 | depleted | 6.68997361 | WD2101_soil_group |
| OTU_2517 | -3.55765768 | 6.936533 | 9.426777 | 0.002138398 | -1 | depleted | 6.14769846 | Vibrionales |
| OTU_1773 | -3.46060023 | 7.454437 | 8.935985 | 0.002796066 | -1 | depleted | 5.87954182 | Verrucomicrobiales |
| OTU_256 | -5.80758792 | 8.779116 | 9.062754 | 0.002608688 | -1 | depleted | 5.94890797 | Verrucomicrobiales |
| OTU_2891 | -3.72544751 | 6.595259 | 9.686569 | 0.001856198 | -1 | depleted | 6.28922509 | Verrucomicrobiales |
| OTU_168 | 7.41251314 | 8.975302 | 11.85276 | 0.000575759 | 1 | enriched | 7.4598216 | unidentified |
| OTU_975 | 5.07712103 | 7.345215 | 9.660559 | 0.001882663 | 1 | enriched | 6.27506792 | unidentified |
| OTU_794 | 5.20436699 | 7.453259 | 9.326535 | 0.00225859 | 1 | enriched | 6.09301441 | unidentified |
| OTU_508 | 4.89889194 | 8.252873 | 11.26817 | 0.000788474 | 1 | enriched | 7.14541093 | unidentified |
| OTU_351 | 6.88073703 | 8.488897 | 9.150428 | 0.002486599 | 1 | enriched | 5.99683948 | unidentified |
| OTU_73 | 4.5936752 | 9.110094 | 9.737298 | 0.001805662 | 1 | enriched | 6.31682815 | unidentified |
| OTU_240 | 7.36152984 | 9.093846 | 12.08052 | 0.000509515 | 1 | enriched | 7.582051 | unidentified |
| OTU_3006 | -4.74968309 | 6.805084 | 11.52314 | 0.000687351 | -1 | depleted | 7.28266543 | unidentified |
| OTU_766 | 4.93249808 | 9.199209 | 14.20331 | 0.000164082 | 1 | enriched | 8.71514603 | unidentified |
| **SL vs SAL** | | | | | | | | |
| OTU_1159 | 6.76520373 | 8.390505 | 14.20939 | 0.000163552 | 1 | enriched | 8.71837971 | Xanthomonadales |
| OTU_1387 | -4.7678801 | 7.005936 | 10.45688 | 0.001221932 | -1 | depleted | 6.707322033 | Xanthomonadales |
| OTU_558 | -2.96570676 | 7.708416 | 7.521475 | 0.006096772 | -1 | depleted | 5.099995761 | Xanthomonadales |
| OTU_2270 | -4.3130793 | 6.85401 | 8.974719 | 0.002737407 | -1 | depleted | 5.900744189 | Xanthomonadales |
| OTU_146 | 5.42486169 | 8.458819 | 7.34905 | 0.00670982 | 1 | enriched | 5.004183119 | Xanthomonadales |
| OTU_731 | -4.69247567 | 7.785978 | 10.92605 | 0.000948216 | -1 | depleted | 6.960928175 | Xanthomonadales |
| OTU_51 | -3.70811281 | 10.177563 | 9.85009 | 0.001698234 | -1 | depleted | 6.378166274 | Xanthomonadales |
| OTU_1748 | 3.88065975 | 7.256899 | 7.409958 | 0.006486383 | 1 | enriched | 5.038050194 | Xanthomonadales |
| OTU_667 | 5.50115821 | 7.581068 | 8.896067 | 0.002857855 | 1 | enriched | 5.857683893 | Xanthomonadales |
| OTU_1076 | 4.0205302 | 7.29852 | 7.181336 | 0.007366584 | 1 | enriched | 4.910801242 | Xanthomonadales |
| OTU_319 | 2.62448146 | 11.255055 | 9.851248 | 0.001697166 | 1 | enriched | 6.37879573 | Xanthomonadales |
| OTU_1369 | -3.44102631 | 8.210118 | 8.121954 | 0.00437324 | -1 | depleted | 5.432251075 | Xanthomonadaceae_bacterium_WWH73 |
| OTU_3334 | -4.65495332 | 6.965379 | 8.752726 | 0.003091397 | -1 | depleted | 5.779132304 | WD2101_soil_group |
| OTU_1687 | -2.85233762 | 7.676033 | 7.684584 | 0.005569451 | -1 | depleted | 5.190458829 | WD2101_soil_group |
| OTU_3460 | -4.37240241 | 7.108289 | 12.28788 | 0.000455908 | -1 | depleted | 7.693218923 | WD2101_soil_group |
| OTU_2558 | -3.49775542 | 6.676469 | 7.274995 | 0.006992107 | -1 | depleted | 4.962973313 | WD2101_soil_group |
| OTU_2517 | -4.07862838 | 6.936533 | 13.0666 | 0.000300608 | -1 | depleted | 8.109703336 | Vibrionales |
| OTU_1276 | -5.94959775 | 7.608442 | 9.84424 | 0.001703643 | -1 | depleted | 6.37498609 | Vibrionales |
| OTU_604 | -5.78676054 | 7.517776 | 18.72743 | 1.50797E-05 | -1 | depleted | 11.10215832 | Verrucomicrobiales |
| OTU_1151 | -4.35342713 | 7.702433 | 10.39806 | 0.001261474 | -1 | depleted | 6.675474109 | Verrucomicrobiales |
| OTU_2335 | -4.20431019 | 6.854485 | 7.70643 | 0.005502447 | -1 | depleted | 5.202562309 | Verrucomicrobiales |
| OTU_109 | 3.36078092 | 10.216192 | 7.514851 | 0.006119236 | 1 | enriched | 5.096318082 | unidentified |
| OTU_454 | 4.51438542 | 8.942558 | 9.425916 | 0.002139402 | 1 | enriched | 6.147228854 | unidentified |
| OTU_226 | 4.04362044 | 9.380343 | 10.20902 | 0.001397553 | 1 | enriched | 6.573032287 | unidentified |
| OTU_975 | 3.64437112 | 7.345215 | 8.407928 | 0.003735882 | 1 | enriched | 5.589771445 | unidentified |
| OTU_1071 | 5.06597839 | 7.310721 | 8.46889 | 0.003612718 | 1 | enriched | 5.623294889 | unidentified |
| OTU_123 | 4.58235463 | 9.502495 | 8.288083 | 0.003990608 | 1 | enriched | 5.523811586 | unidentified |
| OTU_1242 | 4.5451725 | 7.918778 | 13.79414 | 0.000203971 | 1 | enriched | 8.497531085 | unidentified |
| OTU_508 | 4.45431386 | 8.252873 | 14.45123 | 0.000143836 | 1 | enriched | 8.846838892 | unidentified |
| OTU_398 | 3.74250466 | 9.051935 | 10.78863 | 0.001021255 | 1 | enriched | 6.886723071 | unidentified |
| OTU_614 | 4.65403322 | 7.883611 | 8.007204 | 0.004659162 | 1 | enriched | 5.368919748 | unidentified |
| OTU_73 | 3.2898534 | 9.110094 | 8.943554 | 0.002784504 | 1 | enriched | 5.883685392 | unidentified |
| OTU_93 | 7.90906815 | 9.327289 | 15.22351 | 9.55072E-05 | 1 | enriched | 9.256308552 | unidentified |
| OTU_920 | 5.68535532 | 7.662821 | 15.53937 | 8.08047E-05 | 1 | enriched | 9.42347571 | unidentified |
| OTU_382 | 7.09298079 | 8.622973 | 11.24949 | 0.000796451 | 1 | enriched | 7.135345614 | unidentified |
| OTU_2090 | 3.93875432 | 6.871315 | 7.134183 | 0.007562811 | 1 | enriched | 4.884512369 | unidentified |
| OTU_1265 | 5.18582293 | 7.36958 | 7.374352 | 0.006616064 | 1 | enriched | 5.018254656 | unidentified |
| OTU_947 | 5.08119258 | 7.399935 | 9.762354 | 0.001781216 | 1 | enriched | 6.330458732 | unidentified |
| OTU_430 | -5.25980505 | 8.697059 | 12.67538 | 0.000370502 | -1 | depleted | 7.900652769 | unidentified |
| OTU_1443 | -4.908961 | 7.062076 | 13.94718 | 0.00018802 | -1 | depleted | 8.578964049 | unidentified |
| OTU_766 | 4.83248286 | 9.199209 | 20.29645 | 6.63236E-06 | 1 | enriched | 11.92354921 | unidentified |

| Table S4 Top 15 features identified by LEfSe at order level | | | | | | |
| --- | --- | --- | --- | --- | --- | --- |
| Order | P values | FDR | GL | SAL | SL | LDA score |
| Oceanospirillales | 0.001211 | 0.011345 | 278460 | 1443300 | 39342 | 5.85 |
| Bacillales | 0.038637 | 0.086191 | 796100 | 1461700 | 473510 | 5.69 |
| unidentified | 0.000471 | 0.010253 | 839940 | 185800 | 923290 | 5.57 |
| Sphingomonadales | 0.014653 | 0.038631 | 718960 | 185020 | 374210 | 5.43 |
| Flavobacteriales | 0.010217 | 0.031087 | 62086 | 550770 | 69373 | 5.39 |
| Propionibacteriales | 0.009879 | 0.031087 | 341220 | 146610 | 557990 | 5.31 |
| Rhodospirillales | 0.023588 | 0.058633 | 213360 | 103490 | 503440 | 5.3 |
| Solirubrobacterales | 0.005146 | 0.022384 | 291110 | 87076 | 474390 | 5.29 |
| Rhizobiales | 0.038023 | 0.086191 | 481230 | 406670 | 689500 | 5.15 |
| Gaiellales | 0.001608 | 0.011657 | 243500 | 21920 | 252240 | 5.06 |
| Xanthomonadales | 0.00594 | 0.023489 | 192930 | 54188 | 284960 | 5.06 |
| Rubrobacterales | 0.010362 | 0.031087 | 224530 | 45348 | 90456 | 4.95 |
| Burkholderiales | 0.042022 | 0.089168 | 189860 | 11841 | 55504 | 4.95 |
| Pseudonocardiales | 0.000851 | 0.011345 | 167930 | 19507 | 188250 | 4.93 |
| Sphingobacteriales | 0.001954 | 0.012041 | 191420 | 33497 | 65564 | 4.9 |

| Table S5 Contents of active constituents in *C. deserticola* in the three habitats | | | | | | | | |
| --- | --- | --- | --- | --- | --- | --- | --- | --- |
| Code | Habitat | Echinacoside（mg/g） | Cistanoside A（mg/g） | Acteoside（mg/g） | Isoacteoside（mg/g） | 2'-Acetylacteosid（mg/g） | Tubuloside A（mg/g） | Cistanoside F（mg/g） |
| HM1 | Saline-alkali land | 0.82 | 0.55 | 7.44 | 0.54 | 19.47 | 0.44 | 0.43 |
| HM2 | Saline-alkali land | 5.4 | 1.04 | 10.18 | 0.78 | 12.05 | 1.11 | 0.68 |
| HM3 | Saline-alkali land | 3.63 | 1.13 | 5.96 | 0.43 | 9.21 | 2.09 | 0.27 |
| HM4 | Grassland | 7.92 | 1.86 | 8.07 | 0.57 | 4.41 | 0.9 | 2.07 |
| HM5 | Grassland | 50.34 | 2.63 | 9.3 | 0.66 | 4.79 | 2.57 | 3.13 |
| HM6 | Grassland | 25.62 | 5.82 | 9.86 | 1.25 | 5.94 | 0.72 | 1.36 |
| HM7 | Sandy land | 7.78 | 1.34 | 3.17 | 0.45 | 2.03 | 0.4 | 0.31 |
| HM8 | Sandy land | 3.27 | 1.57 | 1.84 | 0.2 | 5.72 | 0.19 | 0.1 |
| HM9 | Sandy land | 1.36 | 0.61 | 1.53 | 0.24 | 5.58 | 0.74 | 0.38 |

Table S6 Ecological factors of the three habitats of *C. deserticola*

| Habitat | District station number | Average annual temperature(℃)  -EF1 | Average annual maximum temperature(℃)  -EF2 | Average annual minimum temperature(℃)  -EF3 | Average annual water vapor pressure(hpa)  -EF4 | Annual precipitation from 20:00 to 20:00(mm)  -EF5 | Annual precipitation from 08:00 to 08:00(mm)  -EF6 | Annual average daily wind speed(m/s)  -EF7 |
| --- | --- | --- | --- | --- | --- | --- | --- | --- |
| SAL | 51133 | 7.742739726 | 14.60630137 | 1.956712329 | 6.84630137 | 0.797808219 | 0.8 | 2.289863014 |
|  | 51238 | 6.875890411 | 13.44054795 | 1.142739726 | 7.671232877 | 0.548767123 | 0.548767123 | 1.470410959 |
|  | 51241 | 6.095068493 | 12.07068493 | 1.266849315 | 5.666027397 | 0.669589041 | 0.672876712 | 2.762739726 |
|  | 51243 | 9.000273973 | 14.01671233 | 4.666027397 | 5.821369863 | 0.326849315 | 0.32739726 | 2.828219178 |
| GL | 51133 | 7.742739726 | 14.60630137 | 1.956712329 | 6.84630137 | 0.797808219 | 0.8 | 2.289863014 |
|  | 51137 | 7.623013699 | 14.08657534 | 2.332876712 | 6.506575342 | 0.832054795 | 0.832876712 | 2.063013699 |
|  | 51145 | 7.172328767 | 14.41342466 | 1.077808219 | 6.869315068 | 0.79260274 | 0.792876712 | 2.125205479 |
|  | 51156 | 4.225205479 | 10.47890411 | -0.891506849 | 5.038082192 | 0.403835616 | 0.405205479 | 2.057534247 |
| SL | 52674 | 5.469041096 | 12.99890411 | -0.82 | 5.605753425 | 0.577260274 | 0.580273973 | 2.737534247 |
|  | 52679 | 8.596712329 | 15.86383562 | 2.047945205 | 6.684931507 | 0.468493151 | 0.46630137 | 1.649315068 |
|  | 52681 | 8.877534247 | 16.4169863 | 2.092328767 | 5.906849315 | 0.30630137 | 0.310684932 | 2.548219178 |
|  | 52784 | 5.793972603 | 12.08054795 | 0.903561644 | 5.622739726 | 0.970958904 | 0.96630137 | 3.449315068 |
|  | 53419 | 8.863013699 | 15.84849315 | 2.748767123 | 6.529589041 | 0.393150685 | 0.389863014 | 2.664931507 |
|  | 53420 | 7.982465753 | 14.88931507 | 1.686027397 | 6.883835616 | 0.373424658 | 0.372328767 | 2.182191781 |
|  | 53512 | 10.17863014 | 16.85561644 | 4.25369863 | 6.05260274 | 0.422191781 | 0.424657534 | 2.745205479 |

Table S7 Redundancy analysis of bacterial communities and ecological factors

| Order Name | Explains % | Pseudo-F | P | Canocial Eigenvalues | Adjusted Explained Variation |
| --- | --- | --- | --- | --- | --- |
| Sphingomonadales | 45.7 | 5.9 | 0.002 | 91.37% | 82.70% |
| Pseudonocardiales | 22.4 | 4.2 | 0.01 |  |  |
| Streptomycetales | 13.6 | 3.7 | 0.038 |  |  |
| Oceanospirillales | 9.7 | 4.5 | 0.016 |  |  |


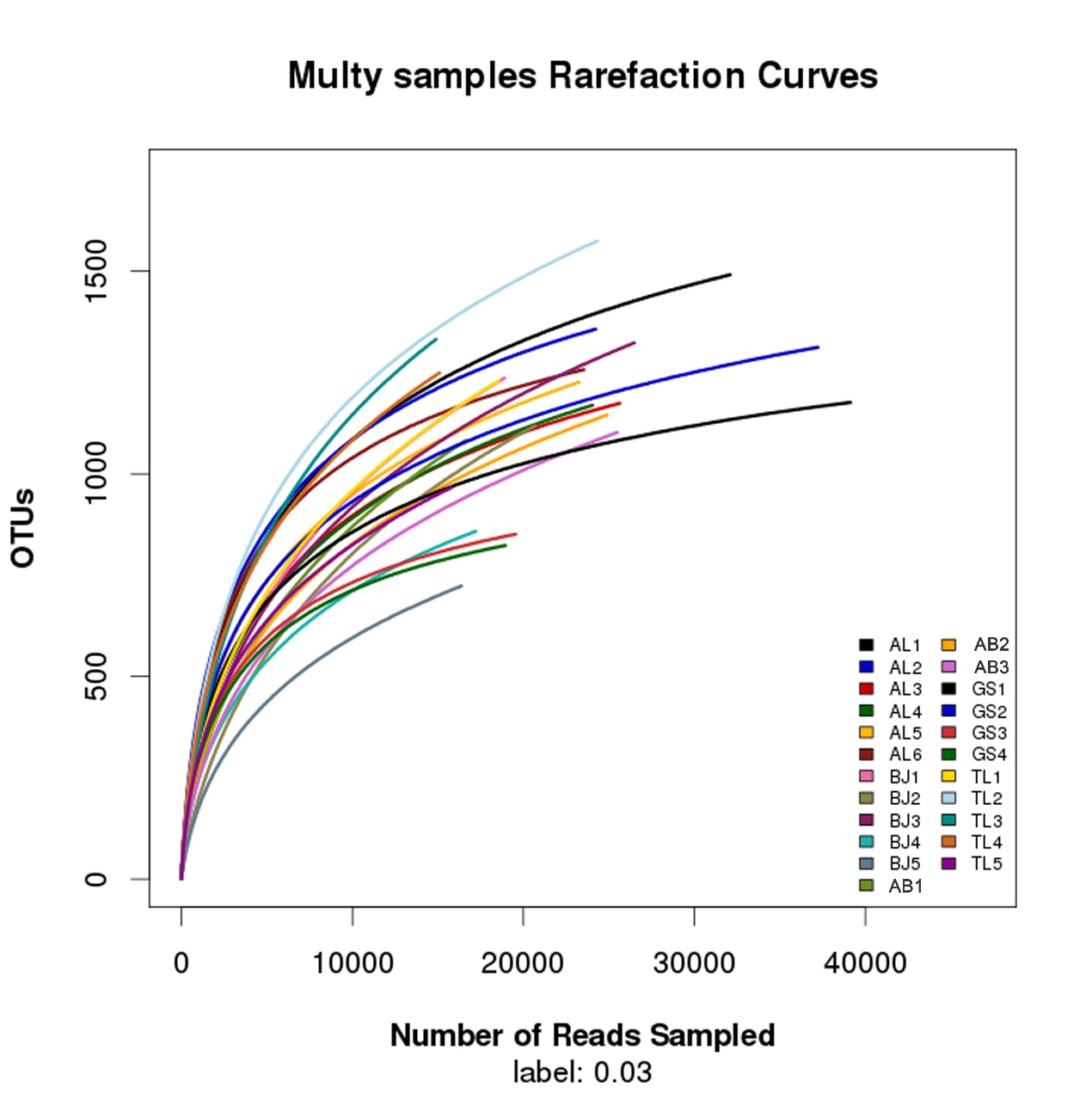


Figure S1 Rarefaction curves showing the observed OTU richness (97% identity). The curves are grouped according to the sample sites. Rarefaction curves for bacterial 16S rRNA gene amplicon libraries.


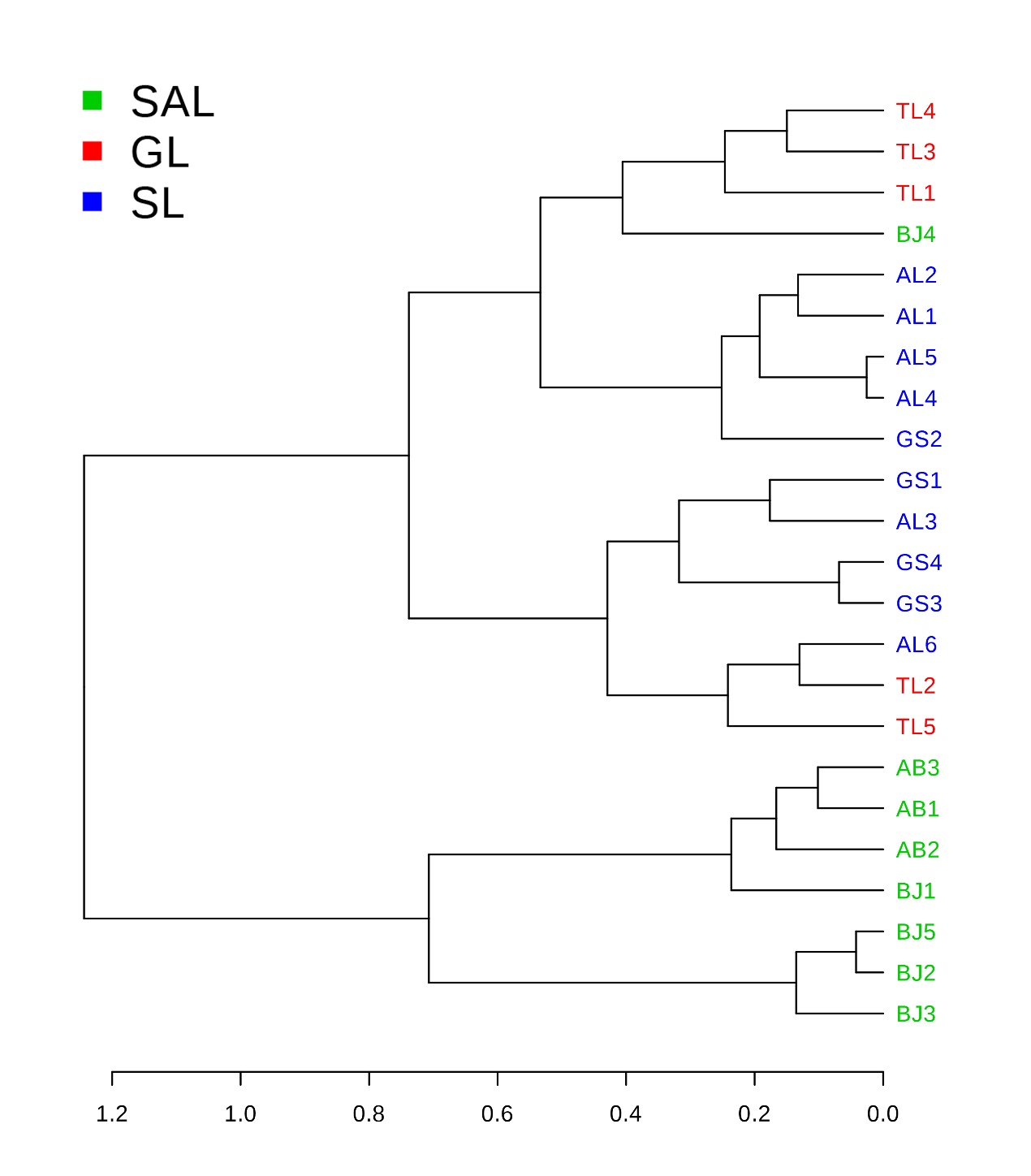


Figure S2 Based on bray distance diversity clustering tree. The length of the branches represents the distance between the samples, and the more similar the samples are the more likely they are to stick together.


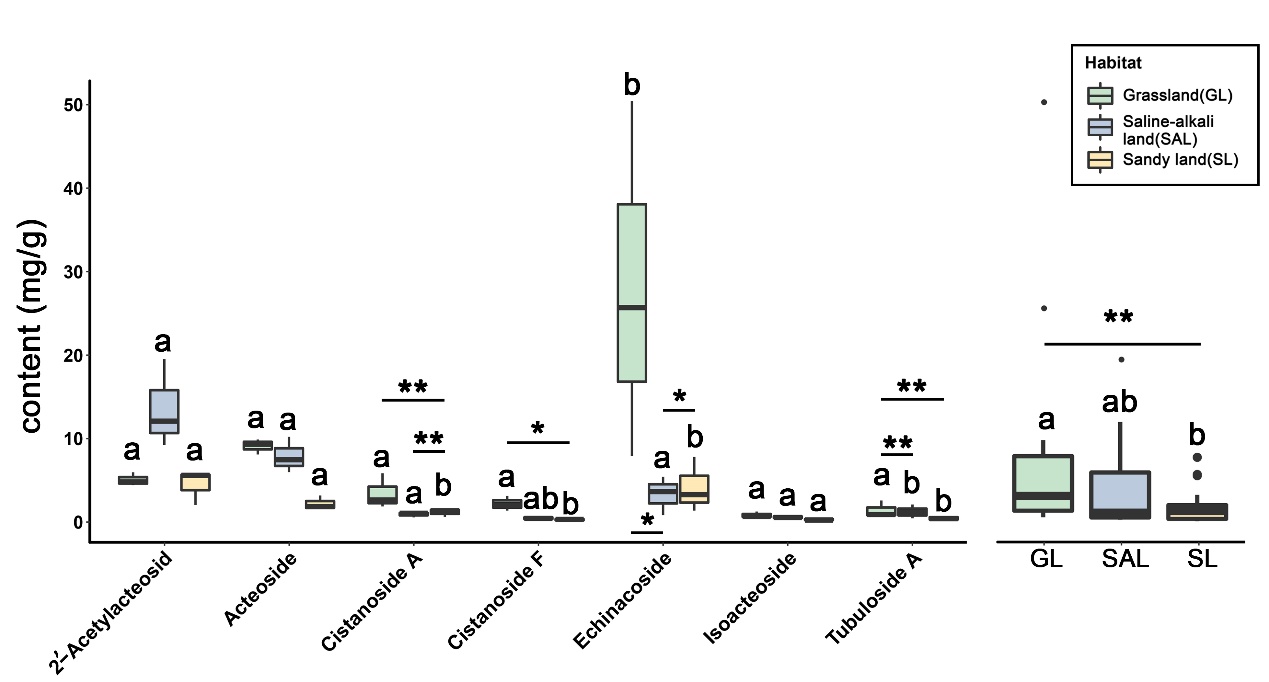


Figure S3 Box diagram of the main active components of *C. deserticola* in the three habitats.* represents P value < 0.05; ** represents P value < 0.01.

1. Corresponding author: Lin-Fang Huang , E-mail : lfhuang@implad.ac.cn;

   Phone: 86-10-57833197; Fax: 86-10-62899700. [↑](#footnote-ref-1)
